# Evaluation of Chemokines MIG and IP-10 as Immunological Biomarkers of Human Visceral Leishmaniasis: A Systematic Review

**DOI:** 10.3390/tropicalmed9090219

**Published:** 2024-09-19

**Authors:** Bruna Eduarda Freitas Monteiro, Elis Dionísio da Silva, Walter Lins Barbosa Júnior, Amanda Virginia Batista Vieira, Roberta dos Santos Souza, Maria Karollyne dos Santos Paiva, Pablo Cantalice Santos Farias, Diego Lins Guedes, Gilberto Silva Nunes Bezerra, Zulma Maria de Medeiros

**Affiliations:** 1 Graduate Program in Health Biosciences and Biotechnology, Aggeu Magalhães Institute, Oswaldo Cruz Foundation (Fiocruz), Recife 50670-420, PE, Brazil; 2 Health and Biotechnology Institute, Federal University of Amazonas, Coari 69460-000, AM, Brazil; elisdionisio@ufam.edu.br; 3 Department of Parasitology, Aggeu Magalhães Institute, Oswaldo Cruz Foundation (Fiocruz), Recife 50670-420, PE, Brazil; walter.lins@fiocruz.br (W.L.B.J.); amanda.bvieira@upe.br (A.V.B.V.); robertassouza@uis.pe.senac.br (R.d.S.S.); 4 Graduate Program in Health Sciences, University of Pernambuco, Recife 50100-130, PE, Brazil; 5 Aggeu Magalhães Institute, Oswaldo Cruz Foundation (Fiocruz), Recife 50670-420, PE, Brazil; karollynemaria1@gmail.com; 6 Department of Genetics, Federal University of Pernambuco, Recife 50670-420, PE, Brazil; pablo.cantalice@ufpe.br; 7 Faculty of Medical Sciences, University of Pernambuco, Recife 50100-130, PE, Brazil; diego.linsguedes@ufpe.br; 8 Life Sciences Center, Academic Center of Agreste, Federal University of Pernambuco, Caruaru 55014-900, PE, Brazil; 9 Department of Nursing & Healthcare, Technological University of the Shannon: Midlands Midwest, N37 HD68 Athlone, Ireland; gilberto.bezerra@tus.ie; 10 Institute of Biological Sciences, University of Pernambuco, Recife 50100-130, PE, Brazil

**Keywords:** visceral leishmaniasis, chemokines, biomarker, MIG, IP-10, systematic review

## Abstract

Visceral leishmaniasis (VL) is a neglected tropical disease that is potentially fatal when untreated. Current diagnostic methods have limitations that contribute to ongoing transmission and poor prognosis. Thus, new tests are needed to provide quick, accurate diagnoses and evaluate clinical progression and treatment efficacy. The monokine induced by interferon-gamma (MIG) and interferon-gamma-inducible protein 10 (IP-10) has been associated with the host susceptibility to VL with potential diagnostic and prognostic purposes. We performed a systematic review using four search databases (Scopus, PubMed, Web of Science, and MEDLINE) to identify studies assessing MIG and IP-10 as potential biomarkers in patients with VL across various clinical conditions. A total of 13 studies were potentially eligible and included in this review. The articles, in general, reveal that the chemokines MIG and IP-10 are elevated in response to infection by *Leishmania* spp., acting on the host’s resistance to the development of the disease. They are associated with asymptomatic conditions and after VL treatment, and this relationship can be observed in both immunocompetent and immunocompromised individuals. Consequently, these chemokines hold relevance in the diagnoses and appropriate management of individuals with VL.

## 1. Introduction

Visceral leishmaniasis (VL), also known as kala-azar, is a severe infectious disease with global distribution, exhibiting a mortality rate of approximately 90% of cases with poor prognosis [1]. It is caused by intracellular protozoa belonging to the *Leishmania donovani* complex: *Leishmania* (L.) *donovani* and *Leishmania* (L.) *infantum*, which are transmitted by infected female sandflies. The parasites are injected into the host, where they infect and multiply within mononuclear phagocytic cells, affecting organs such as the liver, spleen, and bone marrow. Sandflies ingest these infected cells during blood meals, allowing the transmission cycle to continue [1,2,3].

The disease is prevalent mainly in people living under disadvantaged socioeconomic conditions and is linked to risk factors that mainly include malnutrition, poor housing, sanitation, population displacement, and immunocompromised [1,3]. Consequently, VL is characterized as a neglected disease and ranks among the leading causes of mortality when early diagnosis and treatment are not instituted [4].

The identification, treatment, and management of individuals with VL pose significant challenges for control programs [5,6]. This is partly due to the high rate of misdiagnosis, as the clinical symptoms of VL—such as weight loss, hepatomegaly, splenomegaly, and lymphadenopathy—resemble those of other conditions. Additionally, many cases are asymptomatic, leading to undiagnosed individuals silently contributing to the transmission cycle as parasite reservoirs. Another important factor is the limitations of current diagnostic methods in terms of specificity, sensitivity, ease of use, and cost [7,8,9].

Studies reveal that the evaluating levels of monokine induced by interferon-gamma (MIG or CXCL9) and interferon-gamma-inducible protein 10 (IP-10 or CXCL10) may be an excellent strategy to complement existing methodologies used in clinical practice for identifying individuals with VL, particularly asymptomatic ones. It has been observed that MIG and IP-10 levels vary during different stages of infection and are linked to resistance to *Leishmania* spp. This evaluation can provide valuable information on disease progression and treatment outcomes, facilitate appropriate patient management, and prevent relapses and mortality [9,10,11]. 

These molecules interact with the chemokine receptor 3 (CXCR3) and shape the host’s immune response by affecting the types of cells in affected areas, influencing the disease outcome [9,10]. Research shows that the early increase in MIG and IP-10 is linked to the recruitment of CD8+T cells [12,13] and increased IFN-γ expression by natural killer (NK) cells, which influences Th1 differentiation and the activation of pro-inflammatory macrophages [14,15,16,17]. Activated macrophages produce IL-12, which also impacts Th1 differentiation and produces nitric oxide as a defense against the protozoan [17].

Therefore, studies have highlighted the role of these chemokines during active and asymptomatic infection as well as in treated and cured individuals [10,18,19,20,21]. Additionally, IP-10-CXCR3 and MIG-CXCR3 signaling are critical in other intracellular infections. CXCR3-deficient mice, for instance, show increased susceptibility to *Toxoplasma gondii* [22], *Trypanosoma cruzi* [23], and *Chlamydia trachomatis* infection [24].

In this systematic review, we aim to identify potential differences in clinical outcomes between studies for both chemokines. Our investigation examined the median values of the chemokines MIG and IP-10 in asymptomatic and active VL as well as after treatment. By synthesizing and evaluating existing data, we aimed to provide a comprehensive assessment of the comparative roles these two chemokines play in the diagnosis and clinical prognosis of individuals with VL.

## 2. Materials and Methods

### 2.1. Protocol and Registration

This systematic review was conducted according to the 2020 PRISMA—Preferred Reporting Items for System Review and Meta-Analyses [25] [Supplementary File S1]. It was registered by the Prospective International Registry Platform for Systematic Reviews (PROSPERO/National Institute for Health Research—CRD42021241677) [Supplementary File S2].

### 2.2. Data Sources and Search Strategy

A bibliographic survey was carried out by searching for scientific articles from January to May 2024 without any language or publication date restrictions. The studies available in the scientific literature were identified using the following electronic databases: Scopus (Elsevier, Amsterdam, The Netherlands), PubMed (National Center for Biotechnology Information, Bethesda, MD, USA), Web of Science, and MEDLINE (Medical Literature Analysis and Retrieval System Online). The search employed MeSH (Medical Subject Headings) terms in the following combinations: ((Visceral leishmaniasis) AND (MIG or CXCL9)) AND (IP-10 or CXCL10)). MeSH terms were combined using the Boolean operators “AND” and “OR” to access papers with intersections between the different descriptors as well as those containing any of the terms. The terms and combinations were previously tested to identify efficient search for articles. Details about the employed search strategy are available on the PROSPERO platform (accessed on 10 January 2024) [26].

### 2.3. Study Selection and Data Extraction

The articles were selected by two independent reviewers (BEFM and AVBV) using the Rayyan platform [27]. Initially, any duplicate studies from the selected databases were removed. Subsequently, a first screening of the articles was conducted through the reading of titles and abstracts. The second phase of article selection involved reading the full texts in order to perform a new eligibility certification. The inclusion criteria were studies that evaluated levels of the chemokines MIG and IP-10 in patients with VL. Studies that did not address MIG and IP-10 chemokines in human VL and reviews were deemed ineligible, and therefore they were excluded. After the inclusion of eligible articles, we conducted manual searches (hand-searching) using the reference lists of selected articles to find potentially relevant studies that were not identified by the initial search strategy. This step followed the same protocols as before.

Based on inclusion criteria, we extracted and compiled data on the general characteristics of the studies included in this systematic review, such as (a) year of publication, (b) country, (c) endemic species, (d) conditions, (e) study population, and (f) diagnosis, and organized them in Table 1. The values of the quantification of chemokines in pg/mL, sample and method used for quantification, cut-off point, areas under the curve (AUC), sensibility, and specificity provided by the selected studies are presented in Table 2. The median values of the data obtained from the MIG and IP-10 studies in active, asymptomatic, and after VL treatment were calculated using GraphPad Prism v.8.0 software and are organized in Table 3.

### 2.4. Risk of Bias and Quality Assessment

Two independent reviewers (BEFM and AVBV) used the Standard Quality Assessment Criteria for Evaluation of Primary Research Articles from a Variety of Fields [36] to verify the risk of bias and quality of each study included in this review (refer to Table 4). For quantitative studies, this assessment comprises 14 criteria that are scored based on the responses. A score of “2” is given for positive responses, “1” for partial responses, and “0” for negative responses. Criteria that did not apply (N/A) to a specific study were not scored and thus were not included in the score calculation. Scores were expressed as (i) maximum score (28—number of “N/A” × 2), (ii) total score (number of “yes” × 2 + number of “partial” × 1), and (iii) summary score (total score/maximum score). A higher score indicates better study quality and a lower risk of bias [37]. In cases of disagreements between the reviewers, a more experienced third party was consulted (EDS).

## 3. Results

### 3.1. Included Studies

**Figure 1:**
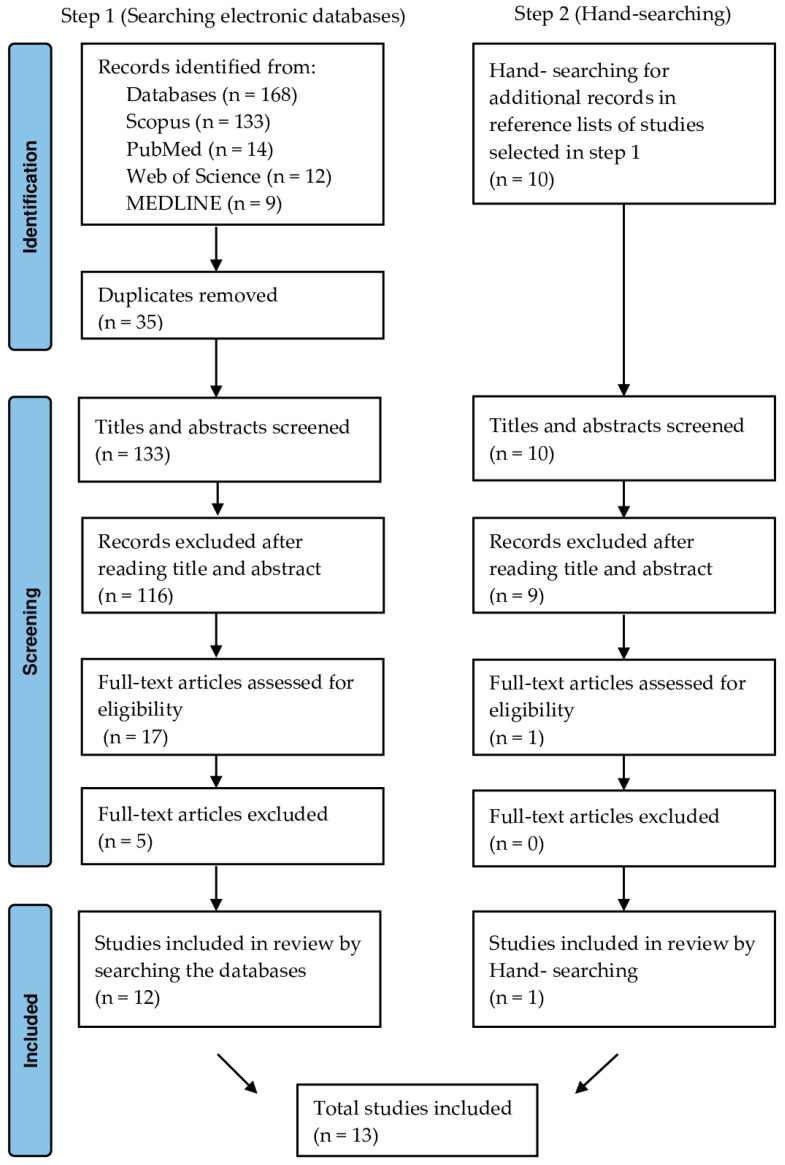
PRISMA (Preferred Reporting Items for System Reviews and Meta-Analyses) flow diagram systematic search and review process.

Based on the search strategy, 168 articles were initially identified across the included databases (Scopus—133, PubMed—14, Web of Science—12, and MEDLINE—9). After removing 35 duplicate articles, 133 remained for titles and abstracts screening. Out of these, 116 studies were excluded for not meeting the eligibility criteria presented in the method section. Thus, a total of 17 articles were selected for detailed analysis through a reading of the full texts, of which 5 were excluded. Therefore, the electronic search generated 12 studies (Step 1), and an additional one was included through a manual search (Step 2). As a result, 13 studies were included in this review. Figure 1 shows the PRISMA 2020 flow diagram of the article selection process.

### 3.2. Description of Studies

**Figure 2:**
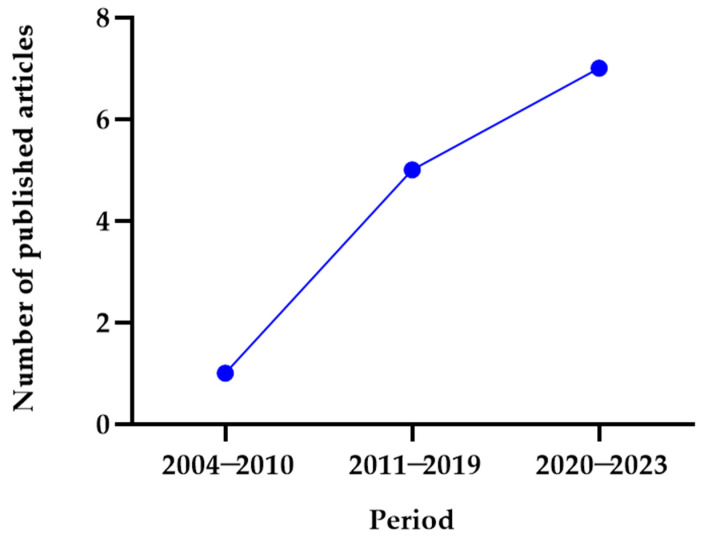
Yearly frequency of studies published on the chemokines MIG and IP-10 in human visceral leishmaniasis.

In this systematic review, we analyzed all studies that evaluated the levels of the chemokines MIG and IP-10 in human VL. The number of publications increased over 19 years, as depicted in Figure 2. Only one article was published between 2004 and 2010 [18], whereas seven studies were published between 2020 and 2023 [10,19,31,32,33,34,35]. Of the total articles included in this review, 8% (1/13) were conducted in India or the United States, 15% (2/13) in Spain and Bangladesh simultaneously, and 23% (3/13) in Spain, Ethiopia, or Brazil (Table 1).

In general, the analyzed population consisted of different groups. Active, identified based on signs and symptoms for VL such as hepatomegaly, splenomegaly, prolonged fever, weakness, lymphadenopathy, and/or weight loss, and confirmation in one or more diagnostic methods, such as parasitological: splenic, bone marrow, and lymph node aspirate; immunological: rK39-ICT (rK39 immunochromatographic test), enzyme-linked immunosorbent assay (ELISA), immunofluorescence antibody test (IFAT); and molecular: nested polymerase chain reaction (Ln-PCR). Asymptomatic, apparently healthy individuals with evidence of exposure/infection and positivity in at least one of the following tests: leishmanin skin test (LST), cell proliferation assay (CPA), rK39-ICT, direct agglutination test (DAT), ELISA, IFAT, conventional PCR (cPCR), real-time PCR (qPCR), and Ln-PCR. Individuals after pharmacological treatment for VL, and negative controls, healthy individuals living or not living in endemic areas for VL, with negative results in the aforementioned tests. In addition to these, five studies evaluated immunocompromised in VL: three analyzed asymptomatic cases of co-infection with HIV [10,21,35], and others studies analyzed cases under autoimmune conditions [32] and in solid organ transplant recipients [33].

Most papers (11/13, 84.6%) investigated the levels of both IP-10 and MIG, identifying significant differences between different groups (active, asymptomatic, and after VL treatment). The chemokine IP-10 was analyzed in all thirteen studies included in this review, and MIG was analyzed in eleven of them [10,18,19,20,21,28,29,30,33,34,35]. Ten studies utilized Cytometric Bead Array (CBA) [10,19,20,21,28,29,30,32,33,35], two employed ELISA [18,31], and one utilized luminex [34] to determine the levels of chemokines. For quantification, some studies used unstimulated plasma [18,28,35] and serum [10,35] samples, while others used soluble *Leishmania* antigen (SLA)-stimulated whole blood [10,35], and peripheral blood mononuclear cell cultures [21,32,34]. In addition, two studies evaluated the stability of the chemokines using Protein Saver cards [19,29].

The majority of the articles included in this systematic review that quantified MIG and IP-10 in SLA-stimulated samples revealed an increase in the levels of the chemokines in asymptomatic individuals [10,20,21,32,33,34,35] and in those treated for VL [19,20,21,29,30,32]. In the study conducted by Ibarra-Meneses et al. [20], significantly higher levels of chemokines were observed in the blood (after stimulation with SLA) of asymptomatic individuals compared to negative controls, with similar mean concentrations of the chemokine IP-10 in asymptomatic cases for *L. infantum* (3303 pg/mL) and *L. donovani* (3406 pg/mL) (Table 2). Regarding the concentration of MIG, small differences were observed between cases of asymptomatic infections by *L. infantum* (892 pg/mL) and by *L. donovani* (604 pg/mL) [20]. 

Ibarra-Meneses et al. [29] found similar results among asymptomatic individuals in areas endemic to *L. infantum* (Spain) and *L. donovani* (Bangladesh). The chemokines MIG and IP-10 were quantified in samples of thawed liquid plasma (TLP) as well as in plasma placed on Protein Saver cards kept at −20 °C (DPS-FZ) and at ambient temperature (DPS-AT). Chemokine levels were higher among samples from infected patients than the negative controls for all three types of prepared samples, and positive correlations between the TLP and DPS-FZ/AT samples for the chemokines were observed in both areas. Quantitative data were provided only among those infected with *L. donovani*, with the values for MIG being 3598 TLP; 205.34 DPS-AT; 241.56 DPS-FZ; and for IP-10: 16,040 TLP; 1435 DPS-AT; and 1416 DPS-FZ [29].

This trend of increased chemokine levels in individuals with asymptomatic VL can also be observed among immunocompromised individuals [10,21,32,33,35] (Table 2 and Table 3). In a study carried out in Spain by Botana et al. [21], through an assay using SLA-stimulated plasma from the WBA and SLA-stimulated PBMC cultures, higher concentrations of IP-10 and MIG were found in asymptomatic VL-HIV individuals when compared to those mono-infected with HIV: MIG: 583.1 pg/mL WBA vs. 5.44 pg/mL controls; IP-10: 2785 pg/mL vs. 2.27 pg/mL; MIG: 13,062 pg/mL PBMC vs. 9.13 pg/mL controls; and IP-10: 1139 pg/mL vs. 0 pg/mL. Similarly, Carrasco-Antón et al. [33], while investigating the levels of chemokines as a means of identifying asymptomatic *L. infantum* infection in solid organ transplant recipients, observed higher levels of both, especially IP-10, compared to those with no-infected subjects (2272 pg/mL vs. 18.22 pg/mL). Although chemokine levels are elevated in immunocompromised individuals, when compared to the median values of these molecules in immunocompetent individuals, a decrease can be observed, associated with their immunocompromised condition (Table 3).

In six studies, the chemokines MIG and IP-10 demonstrated high sensitivity and specificity in identifying asymptomatic individuals, both immunocompetent [20,29,34] and immunocompromised [21,32,33] (Table 2). Analyzing the sensitivity and specificity values provided in the studies, it is observed that high sensitivity is more associated with chemokine quantification performed in WBA than in PBMC [20,21,29,32,33,34]. Considering the infecting species, the chemokine MIG proved to be more sensitive and specific in identifying asymptomatic immunocompetent cases in endemic areas for *L. donovani*. In these cases, the AUC for this chemokine was 1.00, with 100% of infected individuals being identified. However, when considering HIV-immunocompromised individuals, the sensitivity (100), specificity (100), and AUC (1.00) values of the chemokine MIG were able to identify 100% of asymptomatic cases in an endemic area for *L. infantum* [21]. 

In treated VL patients, MIG and IP-10 also showed high sensitivity and specificity (Table 2). Aleka et al. [19] found that IP-10 levels increased significantly one week after treatment (667.90 pg/mL) compared to diagnosis (149.40 pg/mL). Additionally, these levels became even more expressive at the end of treatment (1979 pg/mL). Similarly, for *L. infantum*, IP-10 values at diagnosis were 84.8 pg/mL; during treatment 552.1 pg/mL, and after treatment 1284 pg/mL. At the end of treatment, chemokine levels above the established cut-off point (452.9 pg/mL) were able to identify 92% of cured patients. Ibarra-Meneses et al. [20] also found significantly elevated plasma levels of IP-10 and MIG in blood SLA-stimulated from cured individuals (2638 pg/mL IP-10; 1033 pg/mL MIG) compared to individuals with active VL (270.1 pg/mL IP-10; 123.1 pg/mL MIG). This author also found that the AUC for MIG and IP-10 at 6 months of follow-up after treatment were 0.82 and 0.98, respectively. 

Other studies have shown elevated levels of MIG [18,28,30] and IP-10 [18,28] in active VL. These values were considered significant compared to the after-treatment [18,28,30], asymptomatic groups [18,28], and controls [18,28,30]. Sensitivity, specificity, and AUC values were not provided in any of them, and quantification data in pg/mL were reported in only one article for MIG (11,110.2) and for IP-10 (857.4) [18] in unstimulated plasma samples. The remaining studies presented significant results only through graphs, making it unfeasible to identify the quantification in pg/mL of the evaluated chemokines. 

No study directly suggested a mean value of chemokine levels as applicable biomarkers in different regions where VL is endemic. Based on the values in pg/mL provided, we found differences in the pattern of increase in MIG and IP-10 levels between studies that performed SLA stimulation and those that did not. In summary, for studies with stimulation, there was a trend of increased chemokine levels in the asymptomatic immunocompetent (892 MIG; 3303 IP-10), immunocompromised groups (701.6 MIG; 2528 IP-10), and immunocompetent individuals after VL treatment (1,033 MIG; 1979 IP-10) compared to the active VL immunocompetent group (123.1 MIG; 149.4 IP-10) (Table 3). However, for studies without SLA stimulation, immunocompetent patients with active VL showed higher levels of MIG (11,110.2) and IP-10 (857.4) compared to asymptomatic immunocompetent groups (5649 MIG; <308.7 IP-10) and after VL treatment (7673.5 MIG; 380 IP-10) (Table 3).

### 3.3. Quality Evaluation Criteria

Using the Standard Quality Assessment Criteria for Evaluating Primary Research Articles from a Variety of Fields [36], scores equivalent to the quality of the studies included in this systematic review were obtained, ranging from 60% to 90% (Table 4). The articles that obtained the highest scores—70% [10,18,35], 75% [28], 80% [19,21,32,33], 85% [29], and 90% [20]—provided more complete data as well as greater clarity of information. For those less complete or with missing information, the following scores were applied: 60% [30,31,34]. According to the scores provided, we consider the quality of the studies included in this review satisfactory.

## 4. Discussion

Our systematic review included studies that evaluated MIG and IP-10 in human VL. The publication of these works has been higher in recent years, likely due to increased awareness of the disease, given its high incidence and mortality rate [38,39]. Although this review covered four of the six different regions of the World Health Organization (WHO), only 13 articles on the subject were published in six countries. Given the need for new strategies to optimize the diagnosis, prognosis, and effectiveness of therapeutic interventions identified in the studies, it is highly relevant to investigate biomarkers that can identify individuals with VL and determine clinical progression in endemic areas [9]. These aspects could help in *Leishmania* control, as most infected individuals are not accurately diagnosed and treated [7,9].

The chemokines MIG and IP-10 have been identified as significant biomarkers of VL resistance and correlated with the patient’s clinical progression. It is suggested that such molecules mediate a pro-inflammatory response by regulating the localization and activity of CD4^+^ Th1 cells and effector CD8+ T cells through the CXCR3 receptor to control infections [9]. On the other hand, in active VL, there is susceptibility to infection associated with the anti-inflammatory Th2 profile triggered by the parasite, favoring its multiplying in the host and disease progression [11,31,40]. Despite Th2 anti-inflammatory responses, increased levels of MIG and IP-10, as well as other chemokines including CCL2, CCL3, and CCL5, have been found in active VL [40,41]. This suggests that chemokine responses are not suppressed in this condition, with the absence of response to stimuli from these molecules prevailing [18]. According to some authors, different factors could be linked to this absence of responsiveness, such as elevated levels of cytokines antagonistic to Th1 responses, extensive blocking of Th1 cytokine receptors, or negative regulation of these receptors as a consequence of infection [18,42]. In addition to these, other aspects are discussed, such as the possibility that some immunological processes implicated in this failure are associated with the interference in cell signaling in macrophages, affecting the expression of the transcription factor c-FOS and the inducible nitric oxide synthase enzyme (iNOS). The c-FOS and iNOS play roles in the signaling and activity of macrophages, which are key cells in the immune response against infections, including VL [18,42].

It has been demonstrated that *Leishmania* spp. employs a mechanism to avoid the immune response by cleaving IP-10 with the virulence factor glycoprotein-63 (GP63). This impairs signaling and chemotaxis in CXCR3-dependent cells, which is important for controlling infection as these cells shape the protective immune response [43]. According to some authors, the host positively regulates the transcription of chemokines, and CXCR3-expressing cells are expanded after infection [15]. Singh and Sundar [28] proposed that the increased levels of pro-inflammatory chemokines such as MIG and IP-10 may be a compensatory mechanism for the reduction in the expression of receptors that are negatively regulated during infection. This compromises the recruitment of effector cells to affected tissues and the activation of cells for parasite elimination [28]. This was demonstrated in BALB/c mice with a defect in the positive regulation of CXCR3, which were unable to control *Leishmania* spp. infection [44].

The research by Hailu et al. [18] and Singh and Sundar [28] reported high plasma concentrations of MIG and IP-10 in patients with active VL. However, other studies, such as those by Tasew et al. [31] and others [43], have shown conflicting results. Tasew et al. [31] confirmed reduced levels of IP-10 in whole-blood samples stimulated with *L. donovani* lysates from patients with active VL in Ethiopia. They also noted the absence of production of certain inflammatory molecules such as TNF, IL-6, IL-17, and IL-12p70, with elevated levels of IL-10, which has been linked to impaired T-cell responses. According to the authors [31], the development of the disease is related to the diminished ability to respond to *L. donovani* in terms of cytokine and chemokine production, which can be restored after successful chemotherapy. These divergent results among studies regarding IP-10 levels in active VL, and possibly MIG levels, can be attributed to methodological differences between the studies. For instance, Tasew et al. [31] assessed the levels of the chemokine produced in response to *Leishmania* spp. antigen to combat the disease or infection as a memory response. However, Hailu et al. [18] and Singh and Sundar [28] analyzed the levels of circulating chemokines in response to general inflammation without specifically evaluating the *Leishmania*-specific response.

According to a recent scoping review [7], asymptomatic VL is linked to a Th1 cellular response characterized by elevated levels of pro-inflammatory cytokines and chemokines, including MIG and IP-10. Most studies included in our systematic review suggest that these molecules are important biomarkers of resistance to *L. infantum* and *L. donovani* infection. Therefore, they can be used to monitor and assess the potential risk of developing VL in both immunocompetent and immunocompromised individuals who currently do not show clinically apparent symptoms. For instance, a study by Araújo et al. [34] conducted in the USA found that out of the three chemokines (MIG, CCL2, and IL-8) evaluated, MIG showed the best performance in identifying asymptomatic patients, with an AUC of 0.87 and 100% specificity.

Similarly, MIG and IP-10 have been demonstrated to be reliable markers for asymptomatic VL in immunocompetent individuals in endemic areas of *L. donovani* (Bangladesh) and *L. infantum* (Spain) [20,29]. While their role in inducing an effective immune response against *L. infantum* and *L. donovani* is well-established [17,20], MIG has been shown to be the best biomarker for identifying asymptomatic individuals residing in an endemic area for *L. donovani* (Bangladesh) [20,29], with a sensitivity and specificity of 100%.

It has been documented that the chemokines MIG and IP-10 are primarily induced in response to IFN-γ stimulation, which has been found to be elevated in patients coinfected with *L. infantum* and HIV (VL-HIV) under highly active antiretroviral therapy (HAART) [21]. Regular therapy allows for the maintenance of an asymptomatic specific cellular immune response to *Leishmania* spp. capable of containing parasitemia, influencing the increase in levels of MIG and IP-10, which correlated positively in our previous study [10]. 

On the other hand, Moraes et al. [35] observed a fivefold increase in MIG and IP-10 in HIV patients who had not received prior treatment and were exposed to *L. infantum.* This increase may be due to differences in unstimulated samples. Analyzing the levels of chemokines in samples exposed to SLA allows for a more comprehensive understanding of the immune responses specific to the pathogen [31]. Furthermore, HIV has been shown to modulate the levels of other chemokines linked to IFN-γ, such as CCL2, CCL3, and CCL5 [40,41], further complicating the immune landscape in co-infected individuals. Therefore, comprehensive studies involving a larger number of co-infected individuals, both before and after HAART treatment, as well as those who develop VL, are needed to deepen our understanding of the immune responses in *Leishmania* spp. and HIV co-infection. The immunopathological mechanisms involving both infections are considered complex and not yet fully understood, highlighting the urgent need for further research in this area [45,46].

The chemokines MIG and IP-10 have also proven useful in identifying asymptomatic individuals undergoing organ transplantation and IP-10 for those receiving immunosuppressive treatment for autoimmune diseases [32,33]. In these cases, determining the concentrations of chemokines could be very helpful in controlling leishmaniasis, since immunosuppression can be considered a risk factor for infection activation. Additionally, these patients act as reservoirs for the disease. According to Carrilo et al. [47], the risk of organ recipients developing VL is about 30 times higher than that observed among immunocompetent individuals. Although these individuals are immunocompromised by medications, they can mount a specific Th1 response to the parasite. This has been demonstrated through the lymphoproliferative response to SLA and the strong production of IFN-γ and TNF observed in this group [47].

The chemokines MIG and IP-10 have also been considered useful for evaluating the efficacy of treatment for VL [19,20,32]. The significantly higher production of MIG and IP-10 in treated individuals compared to active VL subjects reveals a cellular hyporesponsive characteristic of active infection, with its restoration occurring after effective treatment [19,20]. Certainly, a long-term immunological memory response would remain in cured patients, with increased levels being detected in patients six and 12 months after treatment [19,48]. The validation of these biomarkers as an alternative test for confirming cure is proposed, as their elevated levels are inversely correlated with parasite load and may reflect parasite elimination [19]. Monitoring these chemokines is suggested for managing VL patients as well as for confirming a cure in individuals with autoimmune diseases, which is crucial for reestablishing immunosuppression in these patients who are often on suspended immunosuppressive treatment [32].

It is important to assess the effectiveness of the treatment used in patient follow-up for the VL elimination program in endemic countries, especially due to the possibility of relapse [49]. For VL-HIV coinfected individuals, without antiretroviral therapy, the rates of relapse are close to 100% even after an effective anti-*Leishmania* therapeutic intervention [50]. In Ethiopia, a meta-analysis found a therapeutic success rate of 83% soon after the end of the treatment and 72% after six months. These results highlight the necessity of continued follow-up care for these patients even after they are considered cured [50]. Additionally, evaluating immunological biomarkers such as the chemokines MIG and IP-10 could provide more definitive data about the effectiveness of treatment and the necessity of secondary prophylaxis. This would lead to increased patient safety, reduced drug exposure, and ultimately lower health service costs [51].

Further investigations into these chemokines and other immunological molecules in SLA-stimulated samples could promote the development and implementation of strategies involving them. According to Botana et al. [21], chemokines could be easily assessed through whole-blood stimulation assays in healthcare centers, particularly at the point of care. Whole-blood assays have shown promise, especially in the sensitivity of chemokines, in identifying asymptomatic immunocompetent and immunocompromised individuals as well as after VL treatment. Additionally, it is easier to use at the point-of-care level compared to the SLA-stimulated PBMC assay [21,33]. Such strategies could help reduce the incidence and prevalence of cases as well as decrease adverse drug events and mortality. Moreover, these chemokines are pertinent to public health since they would encourage a reduction in public spending on hospital admissions and premature deaths by aiding in the identification and proper management of these individuals.

We believe that assessing these chemokines as infection biomarkers in clinical practice for VL could provide excellent support for existing methodologies. Based on studies that evaluated chemokine levels in SLA-stimulated immunocompetent patient samples, we suggest the following median values for MIG (123.1; 892; 1033) and IP-10 (149.4; 3303; 1979) as potential biomarkers for active, asymptomatic, and after VL treatment groups, respectively. For immunocompromised asymptomatic individuals, the suggested values are 701.6 for MIG and 2528 for IP-10. 

### Limitations

The studies included in this systematic review had some limitations, such as (i) the lack of follow-up to observe the outcomes of cases in relation to the variation in the levels of the chemokines MIG and IP-10; (ii) unavailable values in some articles for cut-off points, sensitivity, and specificity of the chemokines when analyzed; (iii) the lack of standardization related to the type of clinical sample, VL diagnoses, and the type of assay used for chemokine quantification; and (iv) the absence of sample stimulation by SLA in some studies, highlighting the need for further research and optimization of a universal cut-off value for the chemokines to define different clinical conditions. 

## 5. Conclusions

This paper covers the last 19 years of studies since the chemokines MIG and IP-10 began to be studied and were suggested as biomarkers for VL. Despite an increase in the number of studies, only 13 have been published. In general, these articles indicate that chemokines MIG and IP-10 increase in response to *Leishmania* spp. infection, acting on the host’s resistance to the development of the disease. Chemokines are associated with asymptomatic conditions and after VL treatment, and this relationship can be observed in both immunocompetent and immunocompromised individuals. It is suggested that MIG and IP-10 can assist in various aspects, including identifying and clinically managing individuals, assessing treatment response, predicting relapse, and indicating a cure. Our work highlights the potential of these molecules in VL. Additional studies in different disease-prevalent areas, including both immunocompetent and immunocompromised individuals, are recommended. The aim is to establish and validate standardized protocols as well as to establish a universal cut-off value for chemokines in the diagnosis and prognosis of the disease. We believe that increased investment in this field will make a significant contribution to the management and eradication of the disease.

## Figures and Tables

**Table 1:** General characteristics of included studies.

Characteristics	[18]	[28]	[20]	[29]	[21]	[30]	[19]	[31]	[32]	[33]	[34]	[35]	[10]
Year of publication	2004	2017	2017	2017	2019	2019	2020	2020	2021	2022	2023	2023	2023
Country	Ethiopia	India	Bangladesh and Spain	Bangladesh and Spain	Spain	Brazil	Ethiopia	Ethiopia	Spain	Spain	United States	Brazil	Brazil
Endemic species	*L. donovani*	*L*. *donovani*	*L. infantum* and *L. donovani*	*L. infantum* and *L. donovani*	*L. infantum*	*L*. *Infantum*	*L. donovani*	*L. donovani*	*L. infantum*	*L. infantum*	*L. infantum*	*L. infantum*	*L. infantum*
Conditions	VL	VL	VL	VL	VL-HIV	VL	VL	VL	Autoimmune disease	Solid organ transplant	VL	VL-HIV	VL-HIV
Study population (n)													
Active	70	41	12 ^a^	-	-	25	13	29	5	-	-	-	-
Asymptomatic	39	-	57 ^a^, 12 ^b^	40 ^a^, 12 ^b^	13	2, 15, 11 *	-	-	20	14	35	14	53
After treatment	33	10	14 ^a^	-	-	10	13	-	14	-	-	-	-
Negative control	12	19	63	33	19	17	-	26	74	36	14	26	17
Diagnosis	Splenic and lymph node aspirate, LST and DAT	Splenic aspirate and ELISA	CPA, rK39-ICT, DAT and qPCR	CPA, rK39-ICT, DAT and qPCR	CPA, rK39-ICT, ELISA, IFAT, and Ln-PCR	Bone marrow aspirate, cell culture, rK39-ICT, ELISA and qPCR	Splenic and bone marrow aspirate	rK39-ICT and DAT	CPA, rK39-ICT, ELISA, IFAT, and Ln-PCR	CPA, rK39-ICT, ELISA, IFAT, and qPCR	ELISA, IGRA, and qPCR	ELISA	rK39-ICT, DAT, ELISA, KAtex, and cPCR

Negative controls: healthy individuals living or not living in endemic areas for VL, with negative results in the aforementioned tests. Visceral leishmaniasis (VL), Human Immunodeficiency Virus (HIV), Leishmanin skin test (LST), Direct agglutination test (DAT), Enzyme-linked immunosorbent assay (ELISA), Cell proliferation assay (CPA), rK39 immunochromatographic test (rK39-ICT), Real-time polymerase chain reaction (qPCR), Immunofluorescence antibody test (IFAT), Nested PCR (Ln-PCR), IFN-γ release assay (IGRA), Latex agglutination test (KAtex), Conventional polymerase chain reaction (cPCR). ^a^ *L. infantum* infection. ^b^ *L. donovani* infection. * Different positivity rates for asymptomatic infection in individuals from an endemic area were obtained in the study [30], being 2/115 for OnSite IgG and Kalazar Detect, 15/115 for ELISA, and 11/29 for qPCR.

**Table 2:** Chemokine quantification values, sample and method used, cut-off, AUC, sensibility, and specificity in active, asymptomatic, and after VL treatment.

References	Chemokine Quantification pg/mL	Sample Used	Method	Cut-Off	AUC	Sensibility (%)	Specificity (%)
[18]	Active						
	MIG: 11,110.2 IP-10: 857.4	Plasma	ELISA	ND	ND	ND	ND
	Asymptomatic						
	MIG: 5649 IP-10: <308.7	Plasma	ELISA	ND	ND	ND	ND
	After treatment						
	MIG: 7673.5 IP-10: 380	Plasma	ELISA	ND	ND	ND	ND
[28]	MIG: ND IP-10: ND	Plasma	CBA	ND	ND	ND	ND
[20]	Active						
	MIG: 123.1 IP-10: 270.1	Stimulated whole blood by *L. infantum*	CBA	ND	ND	ND	ND
	Asymptomatic						
	MIG: 892 MIG: 604 IP-10: 3303 IP-10: 3406	Stimulated whole blood by *L. infantum* Stimulated whole blood by *L. donovani* Stimulated whole blood by *L. infantum* Stimulated whole blood by *L. donovani*	CBA	312.6 138.1 1965 1678	0.96 1.00 0.99 0.98	92.73 100 98.25 91.67	91.84 100 98 93.31
	After treatment						
	MIG: 1033 IP-10: 2638	Stimulated whole blood by *L. infantum*	CBA	176 1142	0.82 0.98	92.31 92.86	81.82 100
[29]	Asymptomatic						
	MIG: 3598 TLP MIG: 205.34 DPS-AT MIG: 241.56 DPS-FZ IP-10: 16,040 TLP IP-10: 1435 DPS-AT IP-10: 1416 DPS-FZ	Stimulated whole blood by *L. donovani*	CBA	ND	1.00 0.96 0.96 0.99 0.99 0.99	100 83.33 83.33 83.33 83.33 83.33	100 92.31 92.31 92.31 92.31 92.21
[21]	Asymptomatic						
	MIG: 13,062 MIG: 583.1 IP-10: 1139 IP-10: 2785	Stimulated PBMC by *L. infantum* Stimulated whole blood by *L. infantum* Stimulated PBMC by *L. infantum* Stimulated whole blood by *L. infantum*	CBA	3,607 299.4 100.9 1179	0.96 1.00 0.90 0.98	84.62 100 84.62 91.67	93.33 100 100 95.45
[30]	Active						
	MIG: ND IP-10: ND	Stimulated whole blood by *L. infantum*	CBA	34 555	ND	ND	ND
	Asymptomatic						
	MIG: ND IP-10: ND	Stimulated whole blood by *L. infantum*	CBA	34 555	ND	ND	ND
	After treatment						
	MIG: ND IP-10: ND	Stimulated whole blood by *L. infantum*	CBA	34 555	ND	ND	ND
[19]	Active						
	IP-10: 84.8 IP-10: 149.40	Stimulated whole blood by *L. infantum* Stimulated whole blood by *L. donovani*	CBA	ND	ND	ND	ND
	After treatment						
	IP-10: 552.1 first week IP-10: 667.90 first week IP-10: 1284 end of treatment IP-10: 1979 end of treatment	Stimulated whole blood by *L. infantum* Stimulated whole blood by *L. donovani* Stimulated whole blood by *L. infantum* Stimulated whole blood by *L. donovani*	CBA	452.9	ND	ND	ND
[31]	ND	Stimulated whole blood by *L. donovani*	ELISA	ND	ND	ND	ND
[32]	Asymptomatic						
	IP-10: ND	Stimulated PBMC by *L. infantum* Stimulated whole blood by *L. infantum*	CBA	ND ND	0.77 0.94	80 90	83.33 91.67
	After treatment						
	IP-10: ND	Stimulated PBMC by *L. infantum* Stimulated whole blood by *L. infantum*	CBA	ND ND	0.81 0.94	78.57 90.91	100 100
[33]	Asymptomatic						
	MIG: 820 IP-10: 2272	Stimulated whole blood by *L. infantum*	CBA	375.3 762.5	0.93 0.96	78.57 93	100 95
[34]	Asymptomatic						
	MIG: ND	Stimulated PBMC by *L. infantum*	Luminex	2072	0.87	71	100
[35]	MIG: ND IP-10: ND	Plasma/Serum	CBA	ND	ND	ND	ND
[10]	MIG: ND IP-10: ND	Serum	CBA	ND	ND	ND	ND

TLP (Thawed liquid plasma), DPS-AT (Protein Saver 903 cards maintained at ambient temperature), DPS-FZ (Protein Saver 903 cards maintained at either −20 °C), PBMC (peripheral blood mononuclear cells), ELISA (Enzyme-linked immunosorbent assay), CBA (Cytometric Bead Array), AUC (The areas under the curve), ND (data not available).

**Table 3:** Median values of the chemokines MIG and IP-10 in active, asymptomatic, and after VL treatment.

Chemokine (pg/mL)	Active	Asymptomatic		After Treatment
	Immunocompetent	Immunocompetent	Immunocompromised *	Immunocompetent
SLA-stimulated MIG	123.1 ^b^	892 (205.3–3598) ^a^	701.6 (583.1–820.0) ^a^	1033 ^b^
Unstimulated MIG	11,110.2 (9986.7–12,022.5) ^c^	5649 (4108.1–8753.4) ^c^	-	7673.5 (5670.1–10,163.7) ^c^
SLA-stimulated IP-10	149.4 (84.80–270.1) ^a^	3303 (1416–16,040) ^a^	2528 (2272–2783) ^a^	1979 (1284–2638) ^a^
Unstimulated IP-10	857.4 (432.0–1628.2) ^c^	<308.7 ^c^	-	380 (172–744) ^c^

For the median values of MIG and IP-10 related to SLA stimulation, only those quantified in WBA were considered due to their sensitivity and specificity. * Quantification data for chemokines in immunocompromised individuals were provided only among asymptomatic cases. ^a^ Data presented as median with the 25th and 75th percentiles in parentheses calculated using GraphPad Prism v.8.0 software. ^b^ Median values without the 25th and 75th percentiles are provided only in reference [20]. ^c^ Median values with the 25th and 75th percentiles are provided only in reference [18].

**Table 4:** Quality evaluation of the included studies.

Criteria	Studies												
	[18]	[28]	[20]	[29]	[21]	[30]	[19]	[31]	[32]	[33]	[34]	[35]	[10]
Question/objective sufficiently described?	1	2	2	2	2	2	2	1	2	2	1	2	2
Study design evident and appropriate?	2	2	2	2	2	2	2	2	2	2	2	2	2
Method of subject/comparison group selection or source of information/input variables described and appropriate?	1	1	2	2	1	1	2	1	2	1	1	2	2
Subject (and comparison group, if applicable) characteristics sufficiently described?	2	2	2	2	2	1	2	2	2	2	2	2	2
If interventional and random allocation was possible, was it described?	N/A	N/A	N/A	N/A	N/A	N/A	N/A	N/A	N/A	N/A	N/A	N/A	N/A
If interventional and blinding of investigators was possible, was it reported?	N/A	N/A	N/A	N/A	N/A	N/A	N/A	N/A	N/A	N/A	N/A	N/A	N/A
If interventional and blinding of subjects was possible, was it reported?	N/A	N/A	N/A	N/A	N/A	N/A	N/A	N/A	N/A	N/A	N/A	N/A	N/A
Outcome and (if applicable) exposure measure(s) well defined and robust to measurement / misclassification bias? Means of assessment reported?	0	0	0	0	0	0	0	0	0	0	0	0	0
Sample size appropriate?	2	2	2	2	1	2	1	2	1	1	1	2	2
Analytic methods described/justified and appropriate?	2	2	2	2	2	2	2	2	2	2	2	2	2
Some estimate of variance is reported for the main results?	2	2	2	2	2	0	2	0	2	2	0	0	0
Controlled for confounding?	N/A	N/A	N/A	N/A	N/A	N/A	N/A	N/A	N/A	N/A	N/A	N/A	N/A
Results reported in sufficient detail?	0	0	2	1	2	0	1	0	1	2	1	0	0
Conclusions supported by the results?	2	2	2	2	2	2	2	2	2	2	2	2	2
Maximum points	20	20	20	20	20	20	20	20	20	20	20	20	20
Total points	14	15	18	17	16	12	16	12	16	16	12	14	14
Summary score (%)	70	75	90	85	80	60	80	60	80	80	60	70	70

0 if the response is ‘no’; 1 if the response is ‘partial’; 2 if the response is ‘yes’; followed by N/A if not applicable.

## Data Availability

No new data were created or analyzed in this study.

## Supplementary Materials

The following supporting information can be downloaded at: https://www.mdpi.com/article/10.3390/tropicalmed9090219/s1, Supplementary File S1: PRISMA 2020 Checklist; Supplementary File S2: PROSPERO/National Institute for Health Research—CRD42021241677.
