# Supplementary material for: Evaluation of Chemokines MIG and IP-10 as Immunological Biomarkers of Human Visceral Leishmaniasis: A Systematic Review
Source: Trop Med Infect Dis. 2024 Sep 19;9(9):219. doi: 10.3390/tropicalmed9090219 (PMC11435945; doi:10.3390/tropicalmed9090219)
Supplement: Supplementary file 1 [file tropicalmed-09-00219-s001.zip › Supplementary File S2.pdf]

Evaluation of MIG and IP-10 chemokines as immunological biomarkers in human visceral leishmaniasis: a systematic review.

To enable PROSPERO to focus on COVID-19 submissions, this registration record has undergone basic automated checks for eligibility and is published exactly as submitted. This protocol has been amended since registration with changes to the PICOS criteria, data extraction, quality assessment, or data synthesis methods. Previous versions of the registration may be viewed for comparison. PROSPERO has never provided peer review, and usual checking by the PROSPERO team does not endorse content. Therefore, automatically published records should be treated as any other PROSPERO registration. Further detail is provided here.

Citation

Elis Dionisio, Bruna Monteiro, Amanda Vieira, Diego Guedes, Zulma Medeiros, Walter Lins Barbosa Júnior, Giberto Bezerra, Pablo Cantalice, Maria Paiva. Evaluation of MIG and IP-10 chemokines as immunological biomarkers in human visceral leishmaniasis: a systematic review.. PROSPERO 2021 CRD42021241677 Available from: https://www.crd.york.ac.uk/prospero/display_record.php?ID=CRD42021241677

Review question [1 change]

Can MIG and IP-10 chemokines be used as immunological biomarkers in human visceral leishmaniasis?

Searches [1 change]

They will be conducted as follows:

(i) A literature search will be conducted in electronic databases such as Web of Science, PubMed, Scopus, MEDLINE, and LILACS;

(ii) MeSH terms with the Boolean operators (OR/AND) will be applied as search terms, as follows: "MIG", "IP-10", "Chemokine", "Biomarker", and "visceral leishmaniasis".

(iii) The search will be limited to original articles without language and time restriction.

Types of study to be included [1 change]

All kind of studies evaluating the chemokines MIG and IP-10 in patients with VL. There will be no language restriction.

Condition or domain being studied [1 change]

Visceral leishmaniasis (VL) is a chronic disease that affects mainly people living in developing countries. After being infected, the human organism produces a great activation of the immune system cells, increasing pro-inflammatory cytokines. The Th1 cellular immune response is associated with resistance to LV, with the production of IFN-γ by macrophages and negatively influencing the parasite replication. However, the cellular response mediated by the Th2 lymphocyte is associated with susceptibility to VL, related to increased cytokine production, and humoral immunity. Besides, the role of monokine-induced-by-IFN-γ (MIG) and protein 10 inducible by IFN-γ (IP-10) was also perceived in

people with disabilities, cured, and asymptomatic VL. Thus, a systematic review is required to analyze the immunological biomarkers MIG and IP-10 in human visceral leishmaniasis.

Participants/population

Studies that evaluated the chemokines MIG and IP-10 in patients with visceral leishmaniasis

Intervention(s), exposure(s)

None. This review is analyzing the biomarkers MIG and IP-10 in patients with VL.

Comparator(s)/control

None. This review is analyzing the biomarkers MIG and IP-10 in patients with VL.

Main outcome(s) [1 change]

Chemokines such as MIG and IP-10 may be useful as immunological biomarkers in patients with VL.

Measures of effect

Percentage of MIG and IP-10 in patients with VL.

Additional outcome(s) [1 change]

Chemokines such as MIG and IP-10 can be useful as immunological biomarkers in identifying active infection, asymptomatic infection, death, recurrence or cure in patients with VL.

Measures of effect

None.

Data extraction (selection and coding) [1 change]

The references will be screened against predetermined eligibility criteria for inclusion in the review. Two investigators will develop a data extraction form (Maria Karollyne and Amanda Vieira) to achieve a good level of agreement between the data extractors. Relevant data will be extracted from the studies selected for inclusion. We will extract data of author, year, type of chemokine studied, and its role as a biomarker.

Risk of bias (quality) assessment [1 change]

The risk of bias assessment will be carried out by two reviewers (Maria Karollyne and Amanda Vieira) and any disagreement will be resolved by a third person (Elis Dionisio).

The quality of the studies will be assessed by the Standard Quality Assessment Criteria for Evaluating Primary Research Papers from a Variety of Fields, which is a measurement tool constituted for 14 items to assess the methodological quality of each study. Each item receives a score according to the answer such as 2 points if the answer is 'yes,' 1 point if the answer is 'partial' or 0 points if the answer is 'no.' For questions not applicable to a specific study they will be marked as 'Not applicable' and not included for calculation. The scores will be expressed in three manners (i) maximum points based on 28 – (2 x number of 'Not applicable), (ii) total points calculated summing the total score obtained for all items and (iii) summary score represented by the total points obtained dividing it by the maximum points. Thus, a higher

summary score means a lower risk of bias followed by better study quality.

Strategy for data synthesis [1 change]

The data extracted from each study will be organized in tables followed by a narrative synthesis evaluating the role of MIG and IP-10 chemokines.

Analysis of subgroups or subsets [1 change]

We will investigate the studies based on characteristics such as the role of MIG and IP-10 chemokines as immunological biomarkers in visceral leishmaniasis patients. Besides, we expect to classify and analyse the biomarkers according to their role in identifying active infection, asymptomatic infection, death, recurrence or cure in patients with VL.

Contact details for further information

Elis Dionisio da Silva

dionisio.elis@gmail.com

Organisational affiliation of the review

Instituto Aggeu Magalhães - Fundação Oswaldo Cruz

https://www.cpqam.fiocruz.br/

Review team members and their organisational affiliations [1 change]

Miss Elis Dionisio. Instituto Aggeu Magalhães - Fundação Oswaldo Cruz

Miss Bruna Monteiro. Instituto Aggeu Magalhães - Fundação Oswaldo Cruz

Amanda Vieira. Universidade de Pernambuco - UPE

Diego Guedes. Centro Acadêmico do Agreste (CAA) - Universidade Federal de Pernambuco - UFPE

Zulma Medeiros. Instituto Aggeu Magalhães - Fundação Oswaldo Cruz

Walter Lins Barbosa Júnior. Instituto Aggeu Magalhães - Fundação Oswaldo Cruz

Giberto Bezerra. Athlone Institute of Technology (AIT)

Pablo Cantalice. Universidade Federal de Pernambuco

Miss Maria Paiva. Instituto Aggeu Magalhães - Fundação Oswaldo Cruz

Type and method of review [1 change]

Diagnostic, Narrative synthesis, Prognostic, Systematic review

Anticipated or actual start date [1 change]

01 January 2024

Anticipated completion date [2 changes]

01 May 2024

Funding sources/sponsors [1 change]

Fundação de Amparo a Ciência e Tecnologia do Estado de Pernambuco - FACEPE, and Presidents Doctoral Scholarship.

Conflicts of interest

None known

Language

English

Country

Brazil

Stage of review [1 change]

Review Completed not published

Subject index terms status

Subject indexing assigned by CRD

Subject index terms

Biomarkers; Chemokine CXCL10; Chemokine CXCL9; Humans; Leishmaniasis, Visceral

Date of registration in PROSPERO

09 April 2021

Date of first submission

10 March 2021

Stage of review at time of this submission [1 change]

Stage	Started	Completed
Preliminary searches	Yes	Yes
Piloting of the study selection process	Yes	Yes
Formal screening of search results against eligibility criteria	Yes	Yes
Data extraction	Yes	Yes
Risk of bias (quality) assessment	Yes	Yes
Data analysis	Yes	Yes

Revision note

Our changes involve updating the current stage of our systematic review and the addition of two new authors.

The record owner confirms that the information they have supplied for this submission is accurate and complete and they understand that deliberate provision of inaccurate information or omission of data may be construed as scientific misconduct.

The record owner confirms that they will update the status of the review when it is completed and will add publication details in due course.

Versions

09 April 2021

09 April 2021

04 August 2022

18 September 2024
